# Utility of Silver-nanoparticles for Nano-fluorimetric Determination of Vancomycin Hydrochloride in Pharmaceutical Formulation and Biological Fluids: Greenness Assessment

**DOI:** 10.1007/s10895-022-02942-1

**Published:** 2022-06-25

**Authors:** Ahmed R. Mohamed

**Affiliations:** Analytical Chemistry Department, Faculty of Pharmacy, Egyptian Russian University, Badr City, Cairo, 11829 Egypt

**Keywords:** Vancomycin hydrochloride, Silver nanoparticles, Spectrofluorimetry, Biological fluids, Eco-scale, Green analytical procedure index

## Abstract

Vancomycin hydrochloride (VANH) is a glycopeptide antibiotic commonly employed in the prophylaxis and therapy of various gram-positive bacterial life-threatening infections. Due to the narrow therapeutic window of VANH, its serum levels should be well-monitored to avoid its toxicity and to optimize its therapy. Herein, an innovative silver-nanoparticles enhanced fluorescence technique was designed for VANH rapid analysis in its pharmaceutical formulation and biological fluids. This technique is based on reinforcement of VANH fluorescence intensity with silver-nanoparticles that were synthesized by a redox reaction between VANH and silver nitrate in NaOH alkaline medium using polyvinylpyrrolidone as a stabilizer. The produced silver-nanoparticles were characterized by using UV–visible spectroscopy where they have an intense absorption maximum at 415 nm and transmission electron microscope (TEM) micrograph where they are spherical in shape with smooth surface morphology and size of 10.74 ± 2.44 nm. The fluorescence intensity was measured at 394 nm after excitation at 259 nm. Under optimum conditions, a good linear relationship was accomplished between the VANH concentration and the fluorescence intensity in a range of (1–36) ng/mL with a limit of detection of 0.29 ng/mL. Greenness assessment was performed using two assessment tools namely; eco-scale scoring and green analytical procedure index revealing excellent greenness of the proposed technique. The proposed technique was validated according to the International Conference on Harmonisation (ICH) recommendations and statistically compared with the reported HPLC method revealing no significant difference concerning accuracy and precision at *p* = 0.05. The proposed technique depended primarily on water as a cheap and eco-friendly solvent.

## Introduction

Silver-nanoparticles (Ag-NPs) have wide-ranging antimicrobial activities besides their prodigious uses in numerous fields especially those related to drug delivery and analysis. In the field of drug delivery, Ag-NPs are utilized to direct the drugs to the diseased tissues consequently, improving the therapeutic efficacy and minimizing the potential side effects of drugs, particularly in the field of chemotherapy [1]. In the field of drug analysis, Ag-NPs are utilized to develop many sensitive and green techniques (Spectrophotometry, Spectrofluorimetry, and Raman spectroscopy) for the determination of drugs at the lowest cost due to their primary dependence on the water as a cheap and green solvent [2–5]. Furthermore, the AgNPs-enhanced fluorescence technique is a promising trend in today's spectrofluorimetric experiments. Fluorescence enhancement is effectively used to improve the techniques sensitivity and detection limits to nano-levels to be more convenient for quantitative drug analysis in miscellaneous matrices at ultra-trace levels [4, 5].

**Fig. 1:**
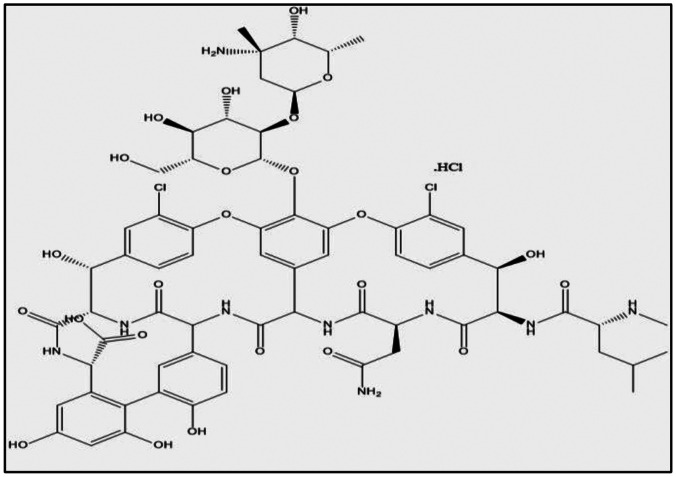
Chemical structure of vancomycin hydrochloride

VANH presented in Fig. 1, is a glycopeptide antibiotic commonly utilized in the prophylaxis and therapy of various gram-positive bacterial life-threatening infections especially those provoked by methicillin-resistant staphylococci [6]. The potency of VANH is due to its capability to impede the peptidoglycan polymers' synthesis of bacteria’s cell wall resulting in bacterial death. Hence, VANH therapy is the first and last resort of defense against serious bacterial infections when other medications have failed due to bacterial resistance or patient sensitivity, especially sensitivity to β-lactam antibiotics [6, 7]. Due to the narrow therapeutic window of VANH (narrow therapeutic window between its effective doses and those at which it produces adverse toxic effects), the under-dosing of VANH leads to insufficient bacterial eradication and developing of antibiotic-resistant bacteria while the over-dosing is primarily correlated with nephrotoxicity and ototoxicity [6, 8]. So, VANH concentration levels should be well-monitored not only in the biological fluids but also in the pharmaceutical commercial products to avoid toxicity or side effects and to optimize VANH therapy. The intravenous LD_50_ values of VANH were 319 mg/kg and 489 mg/kg for rats and mice, respectively [9].

According to the literature survey, some techniques were reported for the estimation of VANH in different matrices including spectrophotometric [10–12], spectrofluorimetric [13–15], electrochemical [7, 16, 17], HPLC [18–26], LC–MS/MS [8, 27–37], capillary electrophoresis [38–41], radioimmunoassay and ELISA-based immunoassay techniques [42–44]. VANH has native fluorescence at 335 nm after excitation at 268 nm according to the previous study [14]. Hence, the purpose of this research is to introduce novel ultrasensitive, simple, cost-effective, and time-saving AgNPs-enhanced fluorescence technique for VANH rapid analysis in its pharmaceutical formulation and biological fluids without interference by the additives of its pharmaceutical formulation or by the matrix of biological fluids yielding satisfactory recovery results comparable to those of the reported HPLC platform [26]. The synthesized Ag-NPs were characterized by using UV–visible spectroscopy and transmission electron microscope (TEM) micrograph. The proposed technique is regarded as a green method for rapid assay of VANH in miscellaneous matrices due to its dependence primarily on the water as a cheap and eco-friendly solvent relative to the other expensive and hazardous organic solvents utilized in most of the reported techniques. Furthermore, the proposed technique’s ultrasensitivity can be adapted for therapeutic monitoring of VANH and studying its bioequivalence in biological fluids.

Greenness assessment of the utilized reagents and procedure was performed to evaluate the proposed technique greenness by using two tools namely; eco-scale scoring and green analytical procedure index revealing excellent greenness of the proposed technique.

## Experimental

### Materials and Chemicals

All chemicals and reagents used during this study were of analytical grade; bi-distilled water was utilized throughout the study.

- The standard of VANH was kindly supplied from Sigmatec Pharmaceutical Industries (Giza, Egypt) with a purity of 99.61% according to the manufacturer’s purity certificate.
- Methanol, acetonitrile, ethanol, 2-propanol, boric acid, phosphoric acid, glacial acetic acid, and acetone (Adwic, Egypt).
- Sodium hydroxide, (5 × 10^–3^ M and 2 × 10^–1^ M) aqueous solutions (Adwic, Egypt).
  —Britton-Robinson buffer solutions (pH 2–12) were prepared by mixing equal volumes of (0.04 M phosphoric acid, 0.04 M acetic acid, and 0.04 M boric acid) solutions subsequently, pH of these solutions was adjusted to the required pH (2–12) using a 0.2 M NaOH aqueous solution [45].
- Silver nitrate, (3 × 10^–3^ M) aqueous solution (Sigma-Aldrich), should be freshly prepared and protected from light during use.
  — A (3×10^-3^ M) AgNO_3_ solution was prepared by dissolving 102 mg AgNO_3_ crystals in 150 mL bi-distilled water into a 200-mL volumetric flask then diluting with bi-distilled water to the mark.
- Polyvinylpyrrolidone, (0.14%) aqueous solution (Sigma-Aldrich).
  — A (0.14%) PVP solution was prepared by dissolving 280 mg PVP powder in 150 mL bi-distilled water into a 200-mL volumetric flask then diluting with bi-distilled water to the mark.
- Human plasma and urine samples were kindly supplied from Zagazig University Hospitals (Sharqia, Egypt), and preserved at –20 °C until the analysis time.

### Pharmaceutical Formulation

Vancomycine^®^ Mylan vial; manufactured by Biologici Italia Laboratories S.R.L. (Milan, Italy); batch number (B1527); labeled to contain 500 mg of VANH.

### Instruments

Fluorescence spectrometer FP-6200 (Jasco, Japan), supplied with a 150-Watt Xe-arc lamp and 1-cm quartz cell was used. At 10 nm, slit widths for both monochromators were set. The fluorimetric measurements were carried out at medium sensitivity using spectra manager software v1.54.

Jasco model V-630 (Japan) double beam UV–visible spectrophotometer with two matched 1-cm quartz cells, connected to an ACER compatible PC with spectra manager II software was utilized to characterize the spectrum of the prepared Ag-NPs.

A JEOL-1010 transmission electron microscope (TEM, Japan) at 80 kV was employed to characterize the size and morphology of the prepared Ag-NPs.

Sonicator (Model WUC-A06H), vortex mixer (Model VM-300), rotary evaporator (Model Scilogex RE 100-pro), and benchtop centrifuge (Model K241R) were utilized.

### Standard Solution

Stock standard solution (100 μg/mL) was prepared by dissolving 10 mg of pure VANH in 70 mL bi-distilled water into a 100-mL volumetric flask using the sonicator for 5 min. Subsequently, the volume was totaled to the 100-mL mark using the same solvent.

Working standard solution (1 μg/mL) was prepared by transferring 1 mL of the stock solution into a 100-mL volumetric flask and then completed to the 100-mL mark with bi-distilled water.

The standard solution was estimated to be stable for 7 days when preserved in the refrigerator as it exhibited no chromatographic or absorbance changes.

## General Procedure

### Preparation of Ag-NPs and Construction of Calibration Plot

By using a micropipette, aliquots of VANH were accurately transferred from its working standard solution (1 μg/mL) followed by the addition of 1.2 mL of AgNO_3_ (3 × 10^–3^ M), 1 mL of PVP (0.14%), and 1.2 mL of NaOH (5 × 10^–3^ M) solutions into a series of 10-mL volumetric flasks. The prepared solutions were heated for 20 min in a water bath that was thermostatically controlled at 90 °C. As a result, Ag-NPs were formed. After cooling the solutions to room temperature, 1 mL of Britton-Robinson buffer solution (pH = 6) was added. Subsequently, the volumes were totaled using bi-distilled water to the 10-mL mark to prepare final concentrated solutions in the range (1–36) ng/mL. The fluorescence intensity values were measured at 394 nm after excitation at 259 nm versus reagent blank handled similarly and concurrently without VANH (Table 1). Each prepared solution was measured three times.

**Table 1:** The optimized analytical parameters required for the determination of VANH by the proposed fluorimetric method

Parameters	Optimized values
λ _excitation_ (nm)	259
λ _emission_ (nm)	394
AgNO_3_ (3 × 10^–3^ M) volume (mL)	1.20
PVP (0.14%) volume (mL)	1
NaOH (5 × 10^–3^ M) volume (mL)	1.20
Heating time (min) at 90 °C	20
Britton-Robinson buffer (pH = 6) volume (mL)	1

The calibration plot was constructed by relating the fluorescence intensity values to the corresponding VANH concentrations in ng/mL followed by computing the regression equation.

### Application to the Pharmaceutical Formulation

From Vancomycine^®^ Mylan vial, an accurately weighed mass equivalent to 10 mg of VANH powder was transferred into a 100-ml volumetric flask containing 70 ml bi-distilled water and subsequently sonicated for 5 min. The solution volume after sonication was totaled to the 100-ml mark using bi-distilled water. From the obtained solution, 1 mL was transferred into another 100-mL volumetric flask and then completed to the 100-mL mark using the same solvent to obtain (1 μg/mL) as a working solution. Finally, the assay was performed as presented before under the general procedure of Ag-NPs preparation to compute the nominal content of VANH in commercial vials and to apply the technique of standard addition.

### Application to Spiked Biological Fluids

To a series of centrifugation tubes, accurately measured aliquots corresponding to 1-mL of thawed drug-free human plasma or urine at room temperature were transferred. Then, different aliquots of VANH working standard solution (1 μg/mL) were spiked into the centrifugation tubes and blended well with 3 mL methanol for 3 min by the vortex mixer for denaturation and precipitation of proteins. For another 20 min at 5000 rpm, the solutions were centrifuged for separation of the precipitated proteins or any insoluble particulate. The clear supernatants were carefully separated and evaporated to dryness by the rotary evaporator. The residues after drying were reconstituted using 3 mL bi-distilled water and transferred directly into a series of 10-mL volumetric flasks. Subsequently, the solutions were handled as declared before under the general procedure of Ag-NPs preparation to obtain final concentrations within the range (1–36) ng/mL. Blank samples were prepared concurrently by the same procedure without VANH. The prepared concentrations of VANH in urine or plasma were computed finally from the calibration plot.

## The Reported Method

An HPLC–UV method [26] was reported for quantitative analysis of VANH in different biological matrices at 215 nm on a Cortecs^®^ C_18_ column using a mobile phase consisting of 20 mM phosphate buffer containing 0.5% v/v of triethylamine (pH 2.5) and a mixture of methanol–acetonitrile (70:30, v/v). The results of the proposed fluorimetric technique and reported HPLC method were statistically compared for evaluating the efficiency of the proposed technique.

## Evaluation of Method Greenness

Two novel approaches were presented to assess the greenness of the proposed method with the reported HPLC method called analytical eco-scale [46, 47] and green analytical procedure index [48].

Analytical eco-scale is a useful semi-quantitative tool used to evaluate any analytical methodology's greenness. It relies on calculating the penalty points of two main parameters of the analytical procedure. The first parameter is known as the reagent parameter that can be computed by concerning amounts, environmental, physical, and health hazards of the used reagents. The second parameter is related to the instrumentation including the instrument's energy consumption, occupational hazards, and amount of waste generated by the device. After computing the penalty points assigned to the aforementioned parameters, the results are subtracted from 100 to obtain the total score required for the greenness assessment. The ideal analytical method is given 100 on the eco-scale score [46, 47]. According to the total score value, the method is considered as an excellent or acceptable, or inadequate green method.

In Table 2, the calculated penalty points for the proposed method were 19 points while were 29 points for the reported HPLC method revealing the excellent greenness of the proposed method. Also, these results confirm the superiority of the proposed method procedure over the reported method due to lower consumption of chemicals and energy as well as lower waste generation.

**Table 2:** Results of eco-scale analysis for the determination of VANH employing the proposed fluorimetric method and the reported HPLC method

Methods	Proposed fluorimetric method	Reported HPLC method [26]
Parameters		
**Reagents**		
Methanol	6	12
Acetonitrile	–	4
Triethylamine	–	6
AgNO_3_	0	–
PVP	0	–
NaOH	2	–
Boric acid	2	–
Phosphoric acid	2	–
Glacial acetic acid	4	–
**Instruments** Spectrophotometer/HPLC		
Energy	0 [≤ 0.1 kWh/sample]	1 [> 0.1 kWh/sample]
Occupational hazard	0	0
Waste	3	6
Total penalty points	Σ 19	Σ 29
Analytical eco-scale total score^a,b^	81	71
	Excellent green analysis	Acceptable green analysis

If the score is > 50, it indicates acceptable green analysis

If the score is < 50, it indicates inadequate green analysis

^**a**^Analytical eco-scale total score = 100–total penalty points

^**b**^If the score is > 75, it indicates excellent green analysis

**Fig. 2:**
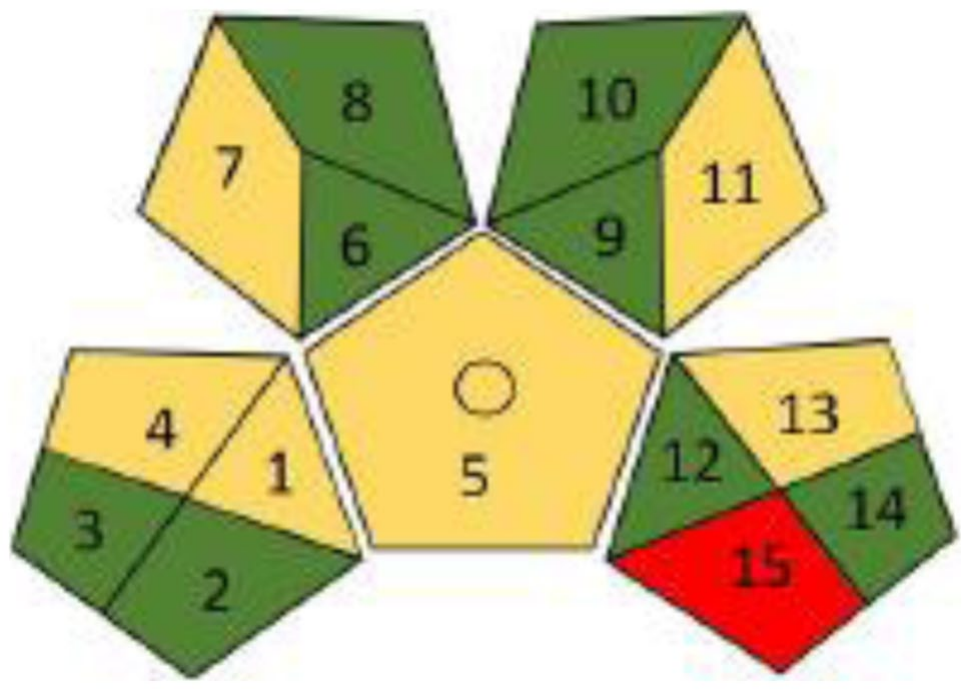
Greenness assessment profile of the proposed fluorometric method using the GAPI tool

**Fig. 3:**
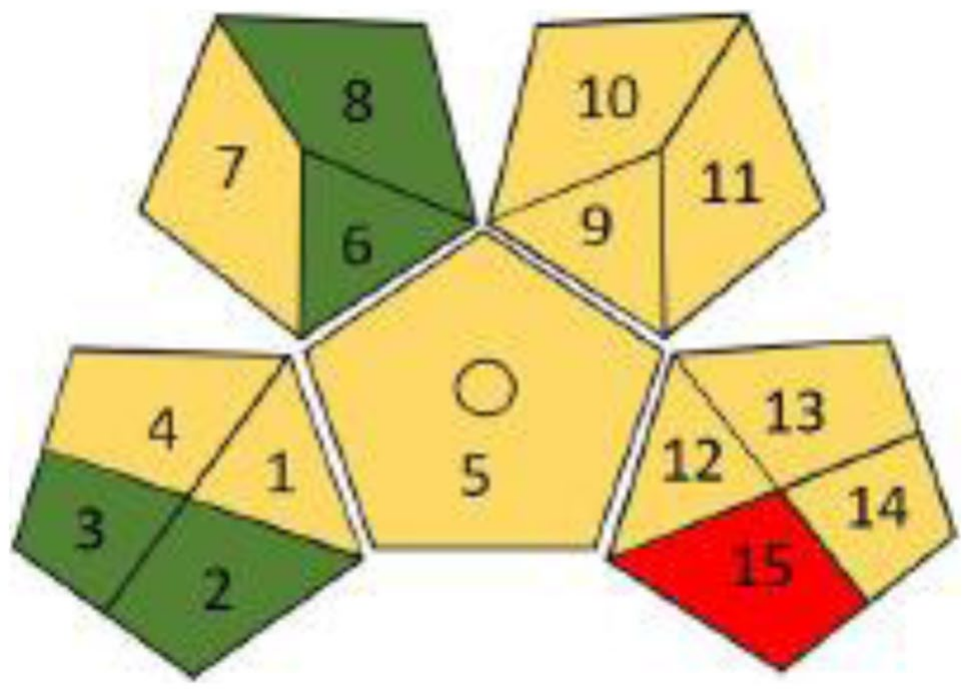
Greenness assessment profile of the reported HPLC method using the GAPI tool

Another tool for greenness assessment of analytical process is green analytical procedure index (GAPI). GAPI is a more advanced tool for greenness assessment. Fifteen-segment pictograms represent different aspects of the analytical process from sample preparation to the final detection. Each segment includes three color-specific codes (green, yellow, or red) to indicate the high, medium, or low environmental impact of each step of the analytical methodology [48]. Figures 2 and 3 showed the greenness assessment profile for the proposed and reported methods’ procedures using the GAPI tool, revealing the superiority of the proposed method procedure over the reported method.

Consequently, the proposed fluorimetric method excels over the reported HPLC method as a greener alternative for the quantitative analysis of VANH in its pharmaceutical formulation and biological fluids.

## Results and Discussion

VANH as a potent glycopeptide antibiotic has an extraordinary role not only in the prophylaxis but also in the therapy of various gram-positive bacterial life-threatening infections. In synthesis or analysis, the majority of laboratories worldwide are moving nowadays towards green chemistry to decrease impacts on the environment and to improve the health safety of analysts. So, a novel ultrasensitive and simple AgNPs-enhanced fluorescence technique was presented for VANH rapid analysis in its pharmaceutical formulation and biological fluids yielding satisfactory recovery results comparable to those of the reported HPLC technique [26].

**Fig. 4:**
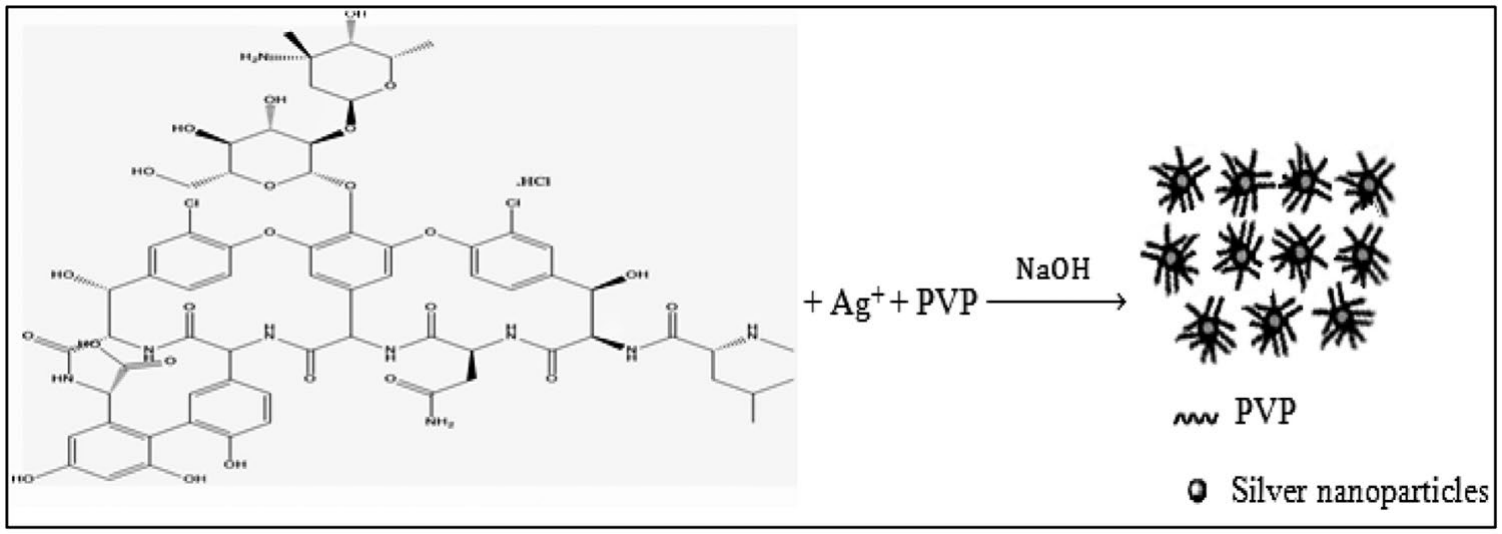
Silver ions reduction by VANH to stoichiometrically equivalent quantity of Ag-NPs

**Fig. 5:**
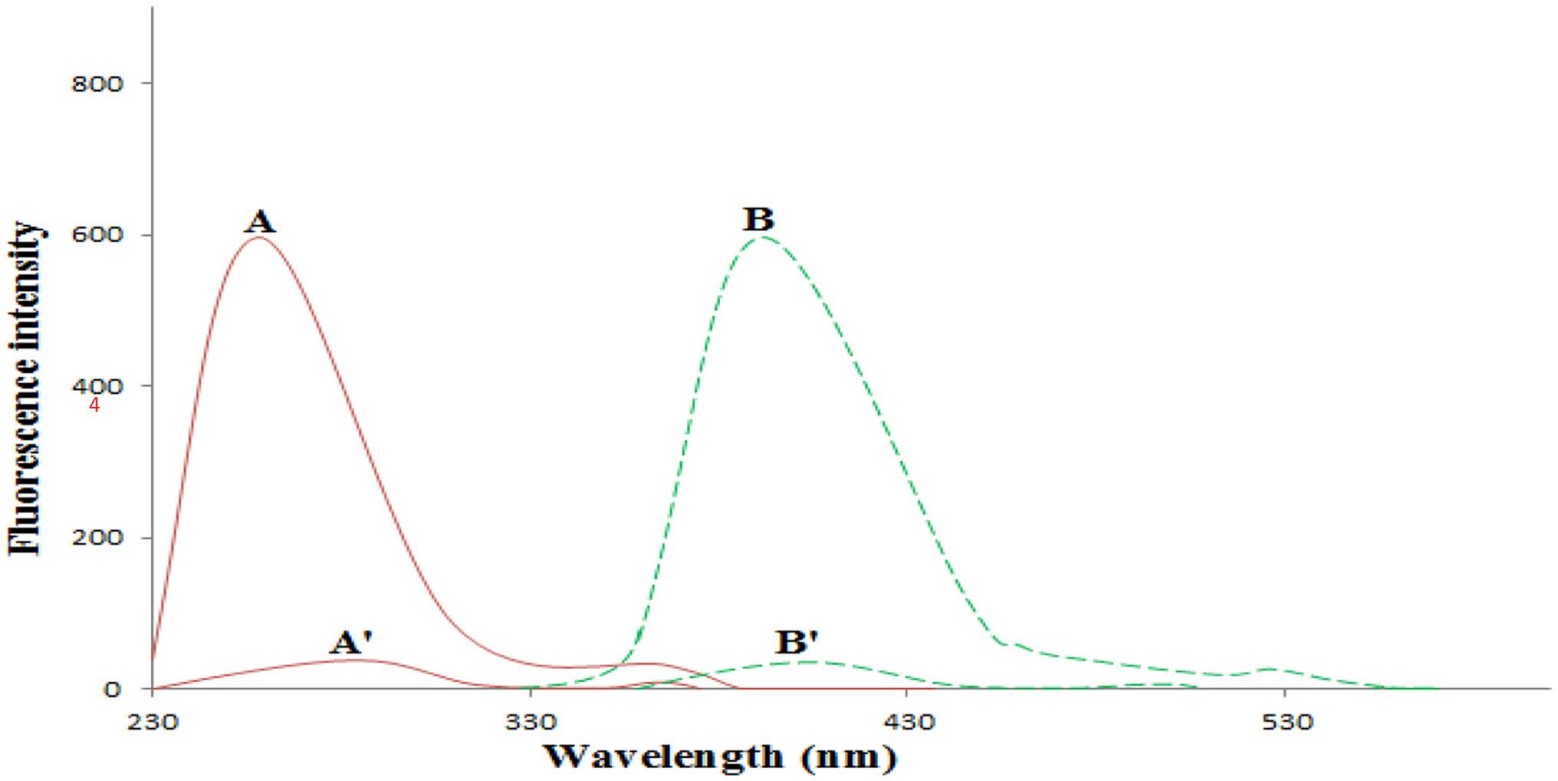
Excitation (**A**) and emission (**B**) spectra of AgNPs-enhanced fluorescent solution of VANH (20 ng/mL) (consists of 0.2 mL of VANH (1 μg/mL), 1.2 mL of AgNO_3_ (3 × 10^–3^ M), 1 mL of PVP (0.14%), and 1.2 mL of NaOH (5 × 10^–3^ M) solutions which were heated for 20 min in a water bath at 90 °C followed by addition of 1 mL of Britton-Robinson buffer solution pH = 6) against the blank reagent (**A**' and **B**') in bi-distilled water as a diluting solvent

In the present effort, the reaction system involved an aqueous AgNO_3_ solution in NaOH alkaline medium with PVP as a stabilizer to prevent agglomeration of Ag-NPs after their synthesis. The addition of VANH as a reducing agent to the reaction mixture led to reduction of the silver ions to a stoichiometrically equivalent quantity of golden yellow Ag-NPs which interacted with the nitrogen atoms of VANH (through electrostatic attraction) [4] producing AgNPs-enhanced fluorescent solution of VANH with high fluorescence intensity and intriguing optical properties (Fig. 4). So, the fluorescence signal of VANH was greatly intensified upon the synthesis of AgNPs-enhanced fluorescent solution. The nano-detection of VANH can be achieved by measuring the fluorescence intensity of the prepared AgNPs-enhanced solution at 394 nm after excitation at 259 nm against the blank reagent (Fig. 5). The fluorescence intensity was observed to be linearly dependent on the concentration of VANH.

**Fig. 6:**
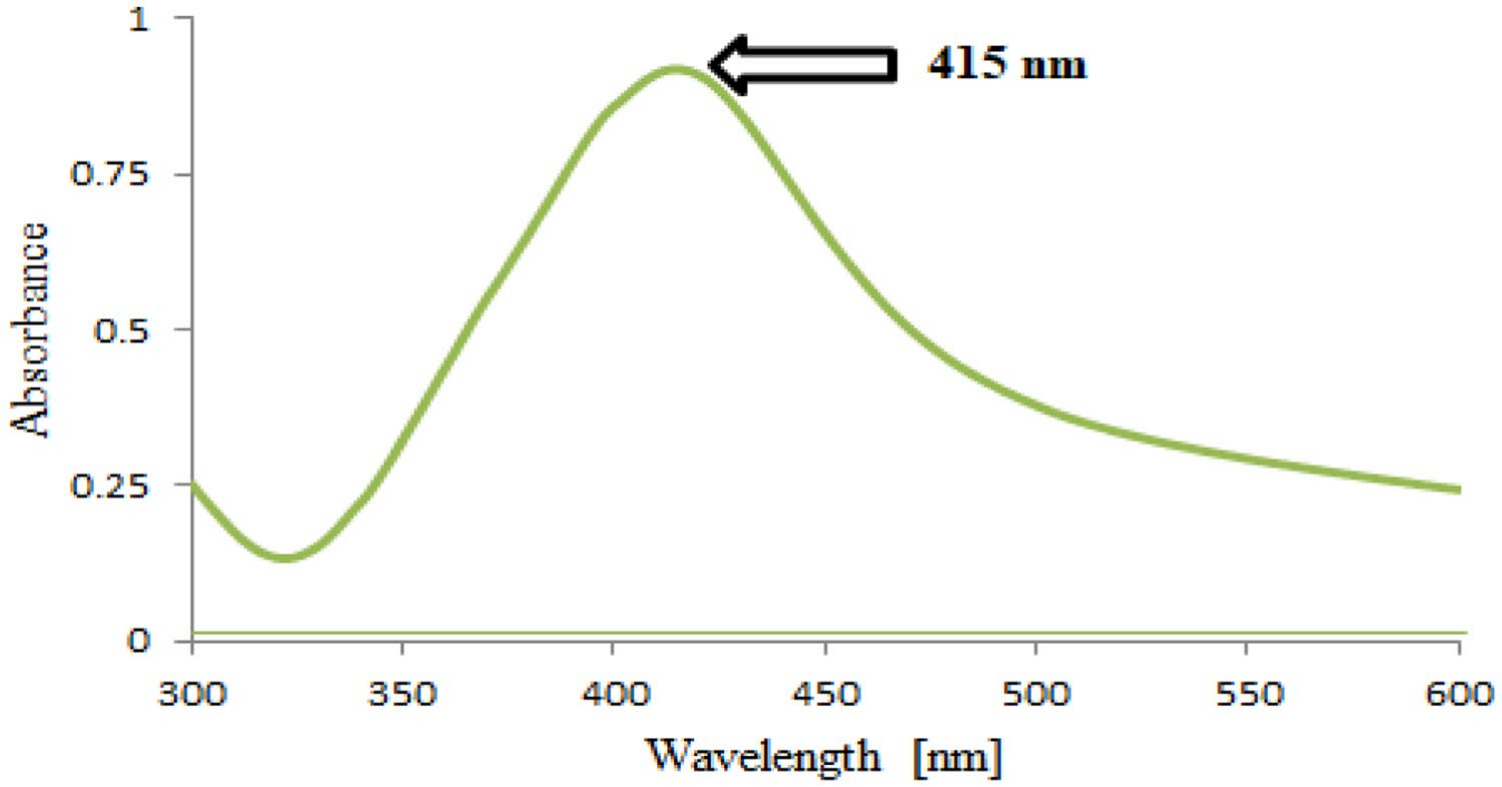
Absorbance spectrum of Ag-NPs developed in presence of VANH (2 µg/mL)

**Fig. 7:**
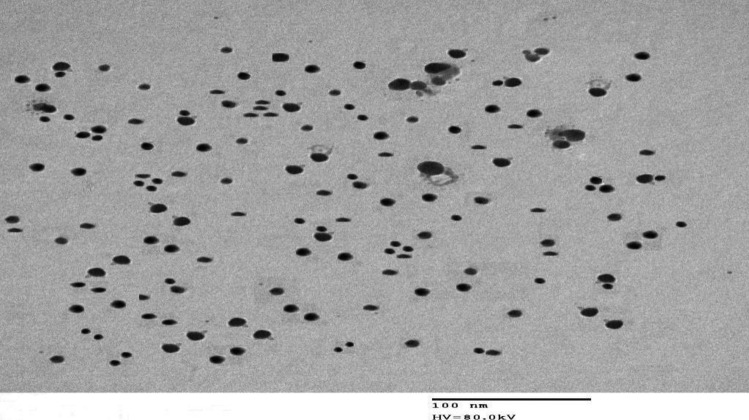
TEM micrograph of Ag-NPs formed in the presence of VANH

The synthesized Ag-NPs were characterized by UV–visible spectroscopy and TEM micrograph. As illustrated in Fig. 6, the Ag-NPs exhibited a characteristic spectrum with an intense absorption maximum at 415 nm due to the surface plasmon excitation. It was observed that VANH absence from the reaction system resulted in absence of any absorption peak in the visible region (400–700 nm). Also, the formation of Ag-NPs in presence of VANH was confirmed as presented in Fig. 7 by the TEM micrograph which reveals that the Ag-NPs were spherical in shape with smooth surface morphology and size of 10.74 ± 2.44 nm.

Unlike conventional fluorometric methods [13, 15], the proposed method is highly sensitive enough to measure VANH concentrations at ultra-trace quantities and consequently, can be adapted for VANH monitoring and studying its bioequivalence in biological fluids. The proposed method is regarded as a green fluorimetric technique appropriate for VANH analysis in miscellaneous matrices at a low cost due to its dependence mainly on water as a cheap and eco-friendly solvent.

### Method Optimization

To obtain optimum results of the proposed technique for the determination of VANH, the following variables were studied:

#### Effect of Concentration and Volume of AgNO3 Solution

**Fig. 8:**
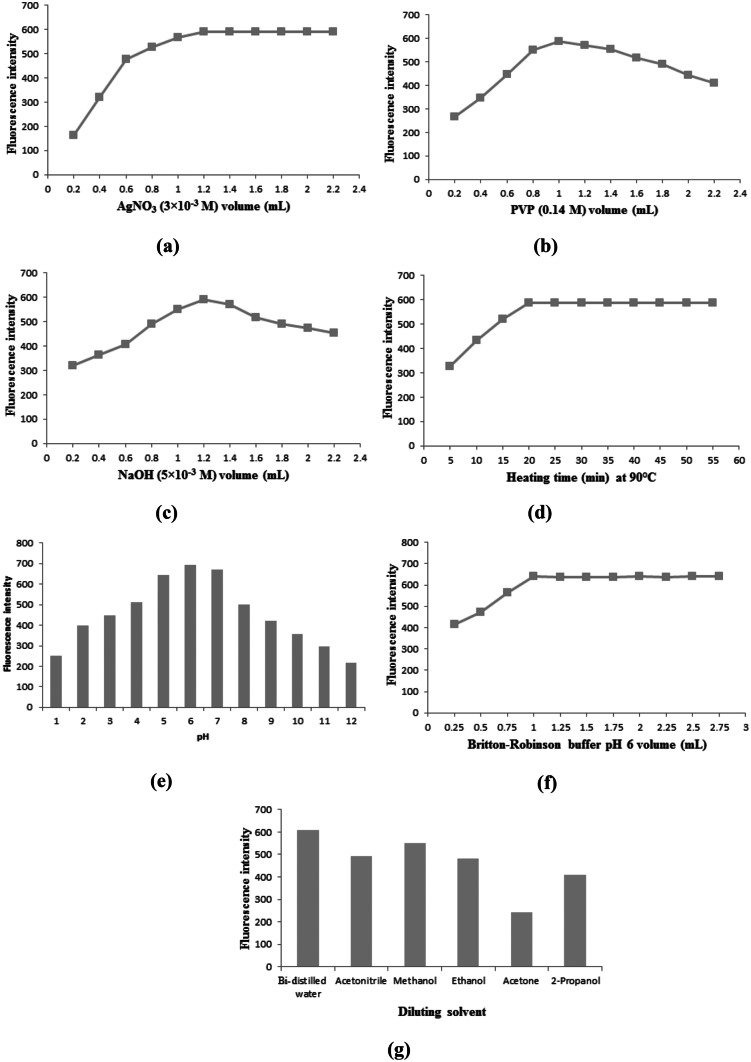
Optimization of experimental variables for VANH (20 ng/mL**)** including: **(a)** Volume of AgNO_3_ (3 × 10^–3^ M) solution. **(b)** Volume of PVP (0.14%) solution. **(c)** Volume of NaOH (5 × 10^–3^ M) solution. **(d)** Heating time effect at 90 °C. **(e)** pH effect. **(f)** Britton-Robinson buffer pH 6 volume. **(g)** Diluting solvent

Several experiments were performed on differently concentrated solutions of AgNO_3_ using the same concentration of VANH in each trial at other optimal reaction conditions (Table 1). As a result, it was observed that AgNO_3_ (3 × 10^–3^ M) solution was the best one for optimum results, after which the increase in the concentration of AgNO_3_ solution resulted in a significant decrease in the fluorescence intensity of AgNPs-enhanced solution due to the formation of AgCl white precipitate. Afterward, different volumes of AgNO_3_ solution (3 × 10^–3^ M) were tried at the same reaction conditions. The results revealed that 1.2 mL was the best volume for optimum results, after which the fluorescence intensity of AgNPs-enhanced solution was almost of the same values with the increase in AgNO_3_ volume (Fig. 8a).

#### Effect of Stabilizer Type, Concentration, and Volume

Ag-NPs are liable to agglomerate during their synthesis. Thus, Ag-NPs were stabilized by one of two stabilizers: electrostatic stabilizers or steric stabilizers to prevent their agglomeration [4]. Electrostatic stabilizers such as sodium citrate, act by adsorption on the nanoparticles’ surface forming an electrical double layer that causes columbic repulsion between the nanoparticles and consequently preventing their agglomeration. While steric stabilizers such as PVP, are characterized by making a protective cap on the nanoparticles’ surface and therefore preventing their agglomeration. In this study, it was observed that using the PVP gave higher fluorescence values than sodium citrate. Thus, PVP was chosen to stabilize Ag-NPs and prevent their agglomeration.

Several trials were performed on different concentrations of PVP solution in a similar way to that of AgNO_3_ solution. As a result, it was noticed that the PVP (0.14%) solution was the best-concentrated one for optimum results, after which the increase in the concentration of PVP solution led to a slight decrease in the fluorescence intensity of AgNPs-enhanced solution. Then, different volumes of PVP solution (0.14%) were tried at the same reaction conditions. The results revealed that 1 mL was the best volume for optimum results, after which the fluorescence intensity of AgNPs-enhanced solution slightly decreased with the increase in PVP volume (Fig. 8b).

#### Effect of Concentration and Volume of NaOH Solution

During the reduction process of silver ions to Ag-NPs by VANH, the H^+^ ions were produced in the reaction medium. Hence, NaOH solution was added to provide enough alkalinity to the reaction medium and to consume the produced H^+^ ions resulting in hastening of the reaction and promoting the reduction process required for Ag-NPs formation. Consequently, the effect of NaOH solution should be well studied by testing differently concentrated solutions of NaOH in a similar way to that of AgNO_3_ solution. After several trials, it was found that NaOH (5 × 10^–3^ M) solution was the best-concentrated one for optimum results, after which the increase in NaOH concentration resulted in a significant decrease in the fluorescence intensity of AgNPs-enhanced solution due to the formation of Ag_2_O black precipitate. Also, different volumes of NaOH solution (5 × 10^–3^ M) were tested at the same reaction conditions. The results revealed that 1.2 mL was the best volume for optimum results, after which the increase in NaOH volume resulted in a gradual small decrease in the fluorescence intensity of AgNPs-enhanced solution (Fig. 8c).

#### Effect of Reaction Temperature and Heating Time

It was observed that the reaction system of the proposed method required heating at 90 °C in a water bath for a certain time to obtain optimum fluorescence values of AgNPs-enhanced solution. After which the increase in reaction temperature resulted in a significant decrease in the fluorescence intensity of AgNPs-enhanced solution due to silver precipitation. Subsequently, different heating times at 90 °C were tested in a similar way to that of the AgNO_3_ solution. It was found that heating at 90 °C for 20 min was the best time for optimum fluorescence intensity results, after which the fluorescence intensity values of AgNPs-enhanced solution remained constant indicating the end of reaction for Ag-NPs synthesis (Fig. 8d). According to the experiment, the fluorescence intensity values remained constant despite heating for 55 min in NaOH solution (5 × 10^–3^ M) indicating the thermal stability of the AgNPs-enhanced fluorescent solution of VANH as a result of using NaOH at a concentration 100 times lower than that reported (5 × 10^–1^ M) in the previous stability study [14].

#### Effect of Britton-Robinson Buffer (pH and Volume)

After several trials, it was found that Ag-NPs can’t be formed in presence of buffer solutions. Consequently, Britton-Robinson buffer solution was added after the formation of Ag-NPs to obtain stable fluorescence intensity values.

So, the effects of pH ranging from 2 to 12 and volume of added buffer ranging from 0.25 to 2.75 mL were studied versus the fluorescence intensity at other optimal reaction conditions (Table 1). It was found that the fluorescence intensity of the AgNPs-enhanced solution gradually increased up to pH 6 at which maximum fluorescence intensity was achieved. At high pH values (> 6), the fluorescence intensity of the AgNPs-enhanced solution decreased gradually as a result of Ag-NPs aggregation under alkaline conditions [49]. So, the choice of pH 6 was crucial to obtain optimum and stable fluorescence intensity values (Fig. 8e). Also, the results revealed that 1 mL of Britton-Robinson buffer solution (pH = 6) was the best volume for optimum results, after which the fluorescence intensity of AgNPs-enhanced solution was almost of the same values with the increase in buffer volume (Fig. 8f).

#### Effect of Diluting Solvent

Upon dilution with different solvents such as bi-distilled water, acetonitrile, methanol, acetone, ethanol, and 2-propanol, bi-distilled water was observed to give the highest fluorescence intensity value (Fig. 8g). Hence, bi-distilled water was the diluting solvent of choice throughout this study.

## Method Validation

According to ICH guidelines [50], the proposed fluorimetric method was validated yielding satisfactory results.

### Linearity and Range

According to the general procedure of the proposed method (Sect. 3.1) and by applying the optimized conditions of the experiment (Table 1), the calibration graph was obtained by plotting the fluorescence intensity values against the corresponding VANH concentrations over the range (1–36 ng/mL). The correlation coefficient and other regression parameters were computed (Table 3). Each prepared concentration of VANH was measured three times.

**Table 3:** Assay parameters for the green analysis of VANH by the proposed fluorimetric method

Parameters	VANH
Concentration range	1–36 (ng/mL)
Correlation coefficient	0.9999
Slope	30.45
Intercept	–6.50
S.D of intercept^a^	2.75
LOD^b^	0.30 (ng/mL)
LOQ^b^	0.90 (ng/mL)
Accuracy	
Mean ± SD	100.89 ± 0.50
RSD%	0.50
Er%^c^	0.33
Intra-day precision^d^	
Mean ± SD	100.60 ± 0.29
RSD%	0.29
Er%^c^	0.31
Inter-day precision^e^	
Mean ± SD	100.61 ± 0.35
RSD%	0.35
Er%^c^	0.25

^a^Standard deviation of intercept

^b^LOD = (SD of the response/slope) × 3.3; LOQ = (SD of the response/slope) × 10

^c^ Relative error percentage

^d^ The intra-day analysis, average of three different concentrations of VANH (10, 20, and 30 ng/mL) repeated three times within the day

^e^The inter-day analysis, average of three different concentrations of VANH (10, 20, and 30 ng/mL) repeated three times in three consecutive days

### LOD and LOQ

To estimate the proposed fluorimetric method sensitivity, LOD and LOQ were computed and listed in Table 3. The exhibited results revealed the ultrasensitivity of the proposed method for VANH determination. For computing of LOD and LOQ, the following equations were utilized:

$${\mathrm{LOD}}\:=\:(3.3\:\times\:\sigma)\;{\mathrm{S}}.$$

$${\mathrm{LOQ}}\:=\:(10\:\times\:\sigma)\;{\mathrm{S}}.$$

Where (σ) is the standard deviation of the response.

(S) is the calibration graph slope.

### Accuracy and Precision

To compute the accuracy and precision at both intra- and inter-day levels, three different standard concentrations of VANH were prepared to cover the low, medium, and higher ranges of the calibration curve (10, 20, and 30 ng/mL) and subsequently analyzed by the proposed method in triplicate. The intra-day analysis (repeatability) was done on the same day while the inter-day one (intermediate precision) was operated on three consecutive days. The obtained accuracy results expressed as mean of percentage recoveries and standard deviation were satisfactory (Table 3). While the calculated values of relative standard deviations (RSD %) didn’t exceed 2% revealing the excellent precision of the proposed method for both intra- and inter-day levels as presented in Table 3, where the percentage relative errors (Er %) were also listed.

### Robustness

To assess the robustness of the proposed method, each parameter of the reaction system was changed separately with a small value keeping the other parameters constant. The results as presented in (Table 4) revealed that the proposed method persisted unaffected by the deliberated small variations in the reaction parameters indicating the robustness of this method.

**Table 4:** Robustness study of the proposed fluorimetric method using pure (20 ng/mL) of VANH

Condition	Results
	Recovery%^a^ ± SD
Optimum condition	100.79 ± 1.02
AgNO_3_ (3 × 10^–3^ M) (1.1 mL)	98.64 ± 0.36
AgNO_3_ (3 × 10^–3^ M) (1.3 mL)	100.16 ± 0.53
PVP (0.14%) (0.9 mL)	98.93 ± 0.37
PVP (0.14%) (1.1 mL)	98.76 ± 0.99
NaOH (5 × 10^–3^ M) (1.1 mL)	98.78 ± 1.03
NaOH (5 × 10^–3^ M) (1.3 mL)	99.17 ± 0.34
Heating time (at 90 °C) (18 min)	98.35 ± 0.33
Heating time (at 90 °C) (22 min)	99.78 ± 0.39
Britton-Robinson buffer (pH = 5.8)	98.46 ± 0.66
Britton-Robinson buffer (pH = 6.2)	99.20 ± 0.62
Britton-Robinson buffer (pH = 6) (0.9 mL)	100.34 ± 1.13
Britton-Robinson buffer (pH = 6) (1.1 mL)	101.27 ± 1.05

^**a**^Mean of three determinations

## Method Application

### Pharmaceutical Application

The proposed method was successfully applied for quantitative VANH analysis in its commercial Vancomycine^®^ Mylan vial. The values of percentage recoveries mean and relative standard deviation presented in Table 5 were satisfactory and in good agreement with the cited drug label claim without pharmaceutical additives interference. Moreover, the tabulated results ascertained the suitability of the proposed method for the routine analysis of VANH in QC laboratories. The proposed method’s validity was also checked by applying the standard addition technique yielding satisfactory results as presented in Table 5.

**Table 5:** Determination of VANH by the proposed fluorimetric method in Vancomycine® Mylan vial and application of standard addition technique

Product	Recovery%^b^ ± RSD	Standard addition			
		Taken (ng/mL)	Added (ng/mL)	Found (ng/mL)	Recovery%^c^
Vancomycine^®^ Mylan^a^	100.64 ± 0.61	10	5	5.06	101.20
			10	9.92	99.20
			15	15.23	101.53
			20	19.91	99.55
			25	25.11	100.44
			Mean ± RSD		100.38 ± 1.01

^a^ Vancomycine^®^ Mylan vial labeled to contain 500 mg of VANH; batch number (B1527)

^b^ Mean of five determinations

^c^ Mean of three determinations

### Biological Fluids Application

According to the pharmacokinetic study of VANH [6], it was found that the VANH C_max_ was 60 μg/mL after a one-hour infusion of vancomycin (1-gm dose), and about 80 to 90% of this dose was recovered unchanged in the urine within 24 h. The attained ultrasensitivity by the proposed method permitted the determination of VANH in spiked human plasma and urine at ultra-trace quantities. Also, the presented satisfying results in Table 6 proved that the proposed method can be utilized not only in the monitoring but also in the pharmacokinetic study of VANH without any interference by the matrix of plasma or urine.

**Table 6:** Determination of VANH by the proposed fluorimetric method in spiked human plasma and urine

Plasma			Urine		
Added (ng/mL)	Found (ng/mL)	Recovery%^a^	Added (ng/mL)	Found (ng/mL)	Recovery%^a^
10	10.13	101.30	10	9.98	99.80
15	15.08	100.53	15	14.81	98.73
20	20.39	101.95	20	19.74	98.70
25	24.73	98.92	25	25.29	101.16
30	30.18	100.60	30	29.62	98.73
Mean ± SD		100.66 ± 1.13	Mean ± SD		99.42 ± 1.08

^a^Mean of three determinations

## Statistical Analysis

The obtained results by the proposed method for VANH analysis in its pure form were statistically compared with those obtained by the reported HPLC method [26]. So, *t-* and F- values were computed and listed in Table 7 where the computed values didn't exceed the theoretical ones. Consequently, no significant differences in results were found between the proposed method and the reported one indicating the high accuracy and precision of the proposed method**.**

**Table 7:** **Statistical comparison between the proposed and reported HPLC** [26] **methods for VANH analysis in its pure form**

Methods	Proposed fluorimetric method	Reported HPLC method [26]
Parameters		
Mean	100.34	99.90
SD	0.59	0.81
N	6	6
Variance	0.35	0.66
Student^'^s *t*-test (2.23)^a^	1.08	–––
F- value (5.05)^a^	0.53	–––

^a^ The parentheses contain the corresponding theoretical *t* and F values at (*P* = 0.05)

## Conclusion

AgNPs-enhanced fluorescence technique was proposed for the determination of VANH at ultra-sensitive levels which depended on enhancing the fluorescence signal of VANH by the formation of Ag-NPs. This proposed technique was found to have higher sensitivity and lower limit of detection than the comparison method. Moreover, it was successfully applied to commercial vials with excellent recovery and good reproducibility. The rapidness and easiness of the proposed method allowed its application for routine VANH analysis in QC laboratories. Also, the proposed method is suitable for in-vitro routine determination of VANH in spiked human plasma and urine in clinical laboratories that seek an economic, sensitive, and environmentally safe method. Accordingly, it is considered a reliable approach for further clinical studies. The presented effort expands the scope of colloidal Ag-NPs application in the analytical field.

## Data Availability

All data generated or analyzed during this study are included in this article.
